# Supplementary figures and images for: Bacterial communities associated with cell phones and shoes
Source: PeerJ. 2020 Jun 9;8:e9235. doi: 10.7717/peerj.9235 (PMC7292020; doi:10.7717/peerj.9235)

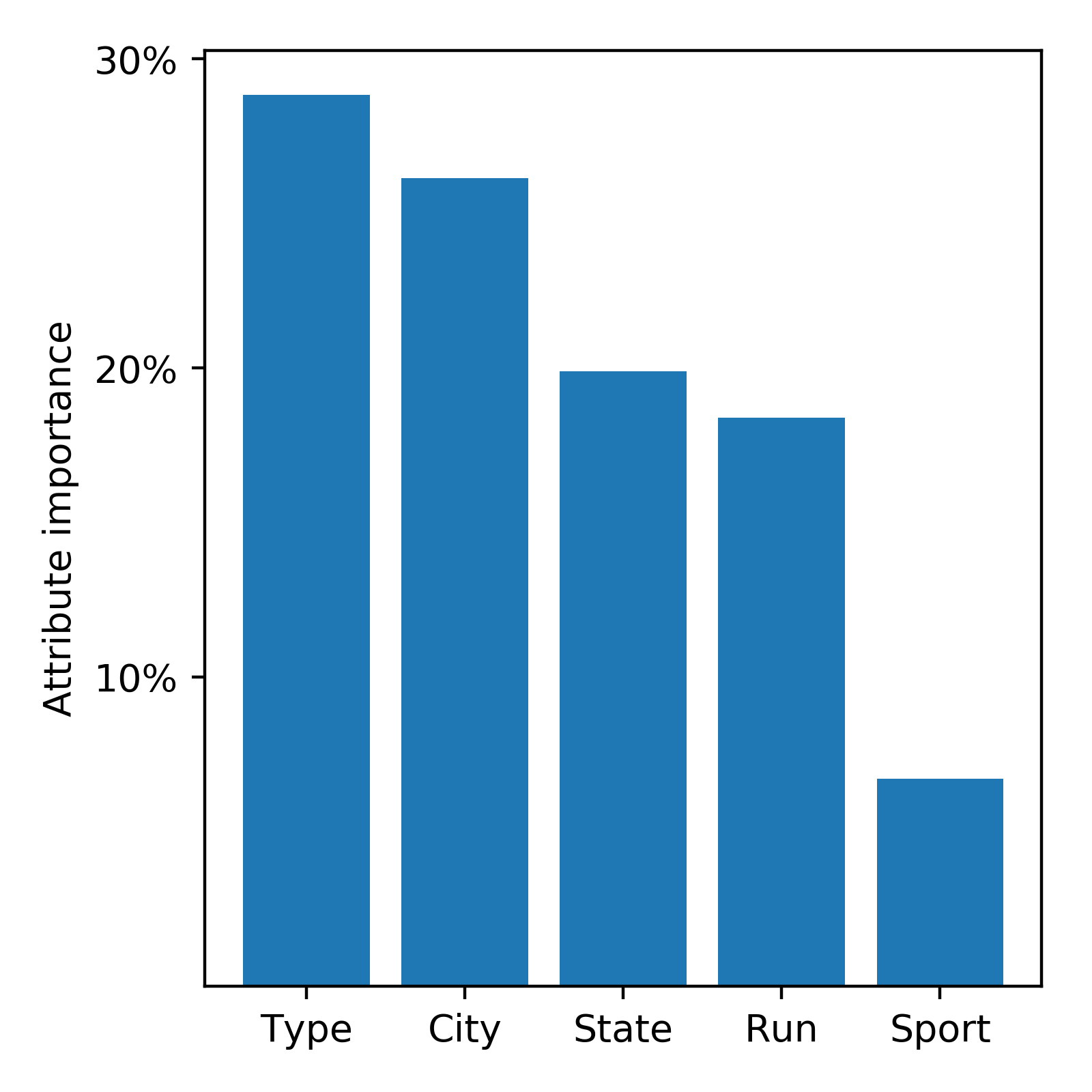

Supplement: Figure S1 [file peerj-08-9235-s001.png]
